# Design of a randomized tobacco cessation trial among FDNY World Trade Center responders in a lung cancer screening program

**DOI:** 10.1186/s13722-025-00598-3

**Published:** 2025-08-05

**Authors:** David G. Goldfarb, Tyrone Moline, David J. Prezant, Matthew P. Bars, Rachel Zeig-Owens, Theresa Schwartz, Madeline F. Cannon, Brandon Vaeth, Julia H. Arnsten, Mayris P. Webber, Shadi Nahvi

**Affiliations:** 1 Montefiore Medical Center, Bronx, NY USA; 2 Albert Einstein College of Medicine, Bronx, NY USA; 3 Fire Department of the City of New York (FDNY), 1 Pierrepont Plaza, 11th floor, Brooklyn, NY 11201 11E-07K USA

**Keywords:** Smoking cessation intervention, Opt-out enrollment, LDCT screening, Biofeedback intervention, World trade center

## Abstract

**Background:**

Cigarette smoking remains the leading preventable cause of death, posing heightened risks for vulnerable populations. World Trade Center (WTC) disaster responders face an elevated burden of respiratory diseases, and despite access to an evidence-based tobacco cessation program, a subset continues to smoke cigarettes. Treatment engagement remains a critical barrier, as many people who smoke fail to enroll in or adhere to programs, particularly when participation requires decisions to actively opt-in to treatment. This randomized controlled trial integrates tobacco treatment into an existing low-dose computed tomography (LDCT) lung cancer screening program and compares the effectiveness of an Enhanced Care intervention with opt-out enrollment and biofeedback to Standard Care with opt-in enrollment and standard treatment.

**Methods:**

The trial includes retired Fire Department of the City of New York (FDNY) responders aged 50 years or older who have a smoking history that satisfies either the National Comprehensive Cancer Network criteria of at least 20 pack-years or a simplified criterion of at least 20 years of smoking. Participants are randomized to either Enhanced Care, featuring opt-out enrollment in tobacco treatment with tailored counseling using biofeedback from chest LDCT and spirometry results, or Standard Care, requiring opt-in enrollment and standard tobacco treatment without biofeedback. Both arms receive a varenicline regimen with 4 weeks of pre-loading. Primary outcomes are treatment enrollment and biochemically verified 7-day abstinence. Factors associated with enrollment and abstinence, including retention, adherence, and quit motivation, will be evaluated.

**Discussion:**

This trial addresses a key gap in tobacco cessation research by testing an innovative intervention for a high-risk occupational cohort participating in LDCT screening. The Enhanced Care model integrates opt-out enrollment, personalized biofeedback, and varenicline preloading to reduce smoking rates and health burdens in FDNY responders. Findings aim to inform scalable cessation strategies for both occupational and general populations, highlighting the need for novel approaches for hard-to-treat individuals who smoke.

**Trial registration:**

This trial was registered at ClinicalTrials.gov under the identifier NCT05997225.

**Supplementary Information:**

The online version contains supplementary material available at 10.1186/s13722-025-00598-3.

## Background

On September 11, 2001 (9/11), firefighters and emergency medical service providers (EMS) from the Fire Department of the City of New York (FDNY) were exposed to dense dust and smoke containing fine particulates and carcinogenic chemicals during the World Trade Center (WTC) disaster rescue/recovery efforts [1, 2]. Over the last two decades, many of these rescue/recovery workers have developed health conditions related to this exposure, including respiratory disease [3] and certain cancers [4]. WTC exposure and smoking are both risk factors for these diseases; while WTC exposure is immutable, smoking is a modifiable risk factor. Smoking rates have declined, in part due to initiatives like the ‘Tobacco Free at FDNY’ Program, which, since 2002, has provided free treatment, including medication and intensive counseling services [5]. Despite access to this free program and the well-publicized risks of cancer and lung function decline, a subset of this cohort continues to smoke [6]. 

Research highlights the significant challenges of engaging individuals who smoke in tobacco treatment and achieving abstinence, both of which are critical for cancer reduction and slowing smoking-related lung function decline [7–9]. Many individuals who smoke face barriers to treatment engagement, often failing to enroll in or adhere to available programs, especially when participation requires an active opt-in. Opt-in models frequently fail to reach individuals who are uncertain or lack sufficient motivation to quit. This is especially problematic for high-risk groups like WTC-exposed responders who smoke, where continued tobacco use exacerbates existing health conditions and contributes to poor overall health outcomes. Addressing these challenges necessitates exploring alternative approaches to more effectively engage people who smoke. Growing evidence suggests that opt-out models, which automatically enroll individuals in treatment, significantly boost participation and quit rates [10–12]. 

Integrating tobacco treatment initiatives into existing healthcare programs, such as low-dose chest computed tomography (LDCT) lung cancer screenings, presents a unique opportunity. While LDCT screening has proven effective in reducing lung cancer mortality, it cannot substitute for the preventive benefits of smoking cessation, which remains the most effective strategy against tobacco-related diseases [13, 14]. Despite declining tobacco use across the U.S. and within FDNY, studies have shown that participants in LDCT screening programs tend to quit smoking at lower rates compared with the general population, potentially due to the misconception that screening reduces the necessity of quitting [5, 15, 16]. Professional guidelines and Centers for Medicare and Medicaid Services (CMS) recommendations endorse including tobacco counseling in LDCT lung cancer screening programs [17]. 

Optimal treatment strategies for enhancing cessation outcomes within LDCT screening programs among people who smoke remain unclear [14, 18]. Most programs offer limited support, often restricted to opt-in Quitline referrals, free, confidential telephone counseling services that offer support for smoking cessation, as well as over-the-counter nicotine replacement therapy (NRT). While pilot studies suggest that using LDCT results as motivational biofeedback holds promise [14, 19, 20], the impact of biofeedback that combines results of LDCT screening and lung function testing via spirometry for promoting smoking cessation remains underexplored. Mixed findings from research on spirometry-based lung function feedback highlight the need to evaluate whether integrating both spirometry and LDCT results into counseling can improve treatment outcomes [21–27]. 

Treatment strategies such as opt-out enrollment, biofeedback, and varenicline with preloading have individually demonstrated effectiveness, but their combined impact when bundled into a comprehensive intervention remains untested. This pilot randomized controlled trial (RCT) in high-risk individuals participating in the FDNY-WTC Health Program (WTCHP) LDCT screening program specifically evaluates the effectiveness of this bundled enhanced care model in improving treatment engagement and quit rates compared to standard care.

## Methods

### Study setting

The FDNY-WTCHP offers responders monitoring exams every 12–18 months, a lung cancer Chest LDCT screening program for high-risk individuals with a 20 pack-year history of smoking, and tobacco treatment services, including free access to medications and counseling. Smoking status and pack-year history have been routinely collected since the 9/11 disaster through standardized self-reported questionnaires administered during regular health monitoring visits as part of the FDNY-WTCHP [28]. The current FDNY-WTCHP treatment protocol includes counseling and nicotine replacement therapies (NRT), with varenicline prescribed as needed. Preloading, where medication is initiated prior to target quit date, is recommended, but not required, to reduce cravings and withdrawal symptoms [29]. 

### Source population

The source population includes retired firefighters and EMS over the age of 50 who are currently participating in the FDNY-WTCHP LDCT Screening Program, a federally funded initiative under the National Institute of Occupational Safety and Health (NIOSH). These individuals were present as responders at the WTC disaster site. To avoid any potential undue inducement, trial participation is limited to retired individuals to ensure their involvement remains independent of decisions regarding work-related inducements, such as promotions or overtime bonuses.

### Analytic study population

FDNY-WTCHP participants are eligible for randomization in this trial if they: (1) self-reported smoking either on their most recent survey or at any time within the last five years; (2) have provided prior consent to be contacted for future health research; (3) meet National Comprehensive Cancer Network (NCCN) [30] Group 2 criteria, which require either a 20-pack-year or a 20-year smoking history [31], with the 20-year cutoff selected for its ability to expand screening eligibility, reduce racial disparities, and provide a simpler, more precise measure of smoking exposure [31]. Patients who are undergoing active cancer treatment, have a history of kidney disease or significantly reduced kidney function (eGFR < 30), or have documented varenicline use in the FDNY-WTCHP medication database within the past year are excluded from the study. Individuals who do not meet the entry criteria could still receive treatment through the FDNY Tobacco Cessation Program but are not included in the study.

**Fig. 1:**
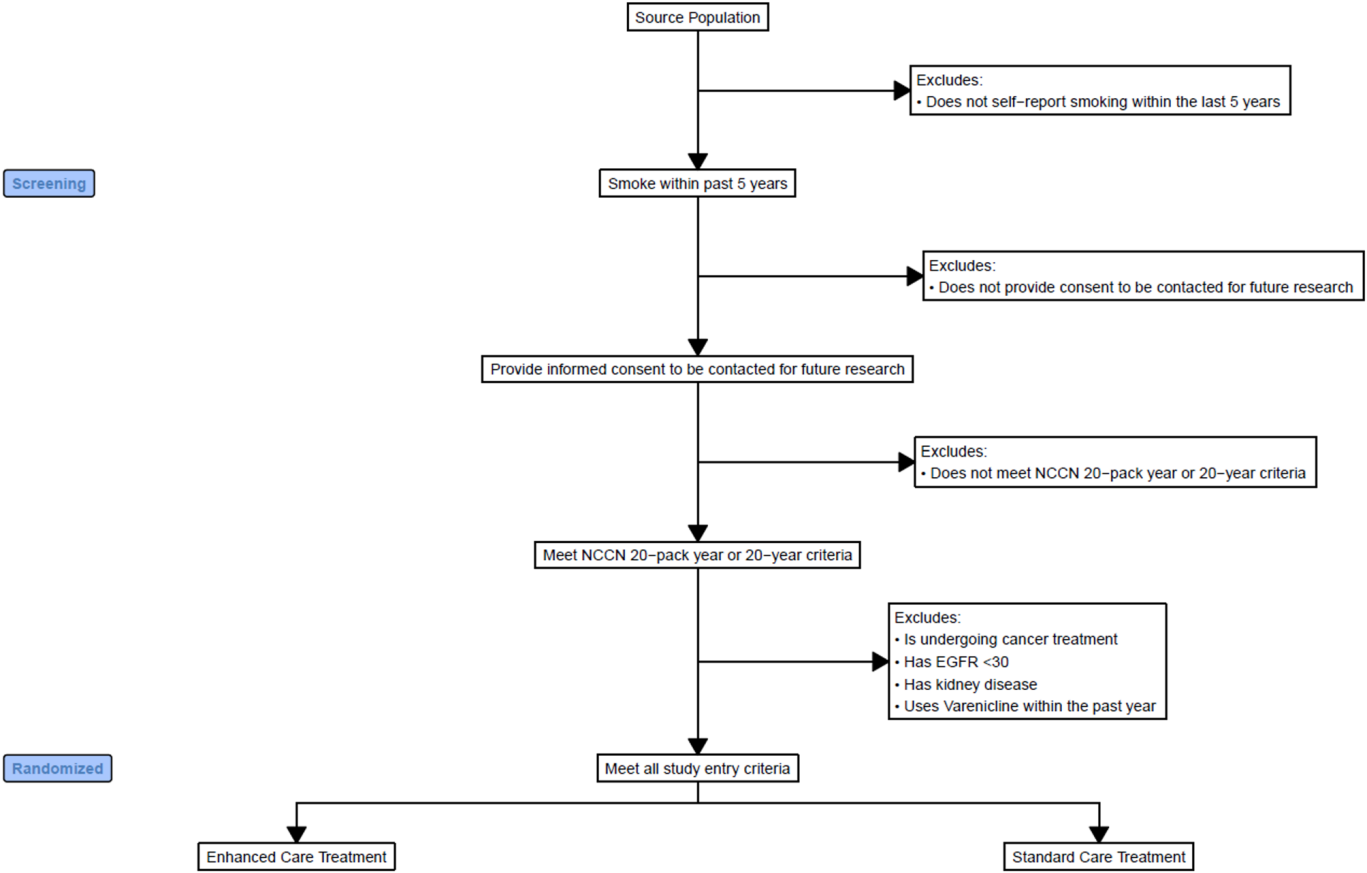
Participant Selection and Study Population Flow. **Footnote**: Abbreviation: EGFR, Estimated Glomerular Filtration Rate. The source population includes participants enrolled in the FDNY WTCHP who are retired and aged ≥ 50 years as on 7/1/2024. Some participants have a 20-year smoking history but do not meet the 20 pack-year criterion

A flow diagram illustrates participant eligibility and randomization is presented in Fig. 1. Inclusion and exclusion criteria are outlined in Table 1. Following eligibility confirmation, participants are assigned to either Enhanced Care or Standard Care using a 1:1 computer-generated randomization process in a blinded fashion. At the time of writing this manuscript, all preparatory procedures, including randomization, have been finalized, and participant recruitment is actively in progress.

**Table 1:** Inclusion and exclusion criteria

Inclusion criteria
• Enrolled in the FDNY World Trade Center Health Program (WTCHP)
• Retired
• Age ≥ 50 years
• Self-reported current cigarette smoking
• Smoking history of: o ≥ 20 pack-years (NCCN criteria) OR o ≥ 20 years of smoking duration
**Exclusion criteria**
• Impaired renal function (eGFR < 30 or diagnosed kidney disease)
• Undergoing active cancer treatment
• Following enrollment into the study: o Use of varenicline within the past year o Moderate or high suicide risk (as assessed by Columbia-Suicide Severity Rating Scale, C-SSRS) o Alcohol use disorder (as assessed by Alcohol Use Disorders Identification Test, AUDIT score > 15) • History of adverse effects or intolerability to varenicline • Clinical instability (psychiatric hospitalization within the past year, decompensated cirrhosis, serious cardiovascular disease, history of seizure disorder)

### Participant recruitment

Figure 1 illustrates the flow of participants from the FDNY WTCHP source population to randomization. All eligible participants meeting inclusion criteria are randomized in a 1:1 ratio into Enhanced Care (opt-out) and Standard Care (opt-in) arms. Recruitment letters are mailed and emailed following randomization. For the Enhanced Care group, the recruitment letter informs participants of their selection for opt-out enrollment, specifying all the clinical services offered as part of the study. One month following the mailing of the letters, the research team initiates proactive outreach by phone to explain the study procedures, risks, and benefits, and to conduct the informed consent process. Participants in the Enhanced Care group are informed that clinical services would begin unless they decline participation. Participants who decline to participate or cannot be reached after three attempts will be excluded from the study without further contact.

For the Standard Care (opt-in) group, the recruitment letter explains that participants have been randomly selected to participate in this study, instructing them to contact the research team if interested and to enroll into the study to access the offered clinical services. Only those who actively reach out to the research team will be included in the study with no further study contact.

### Informed consent

After randomization, participants complete an informed consent, where study procedures, risks, and benefits are explained, emphasizing voluntary participation with no impact on pensions, compensation, or benefits. Consent is provided electronically or verbally (documented and confirmed by letter) for those with platform access challenges, ensuring transparency and engagement.

### RCT study design

**Fig. 2:**
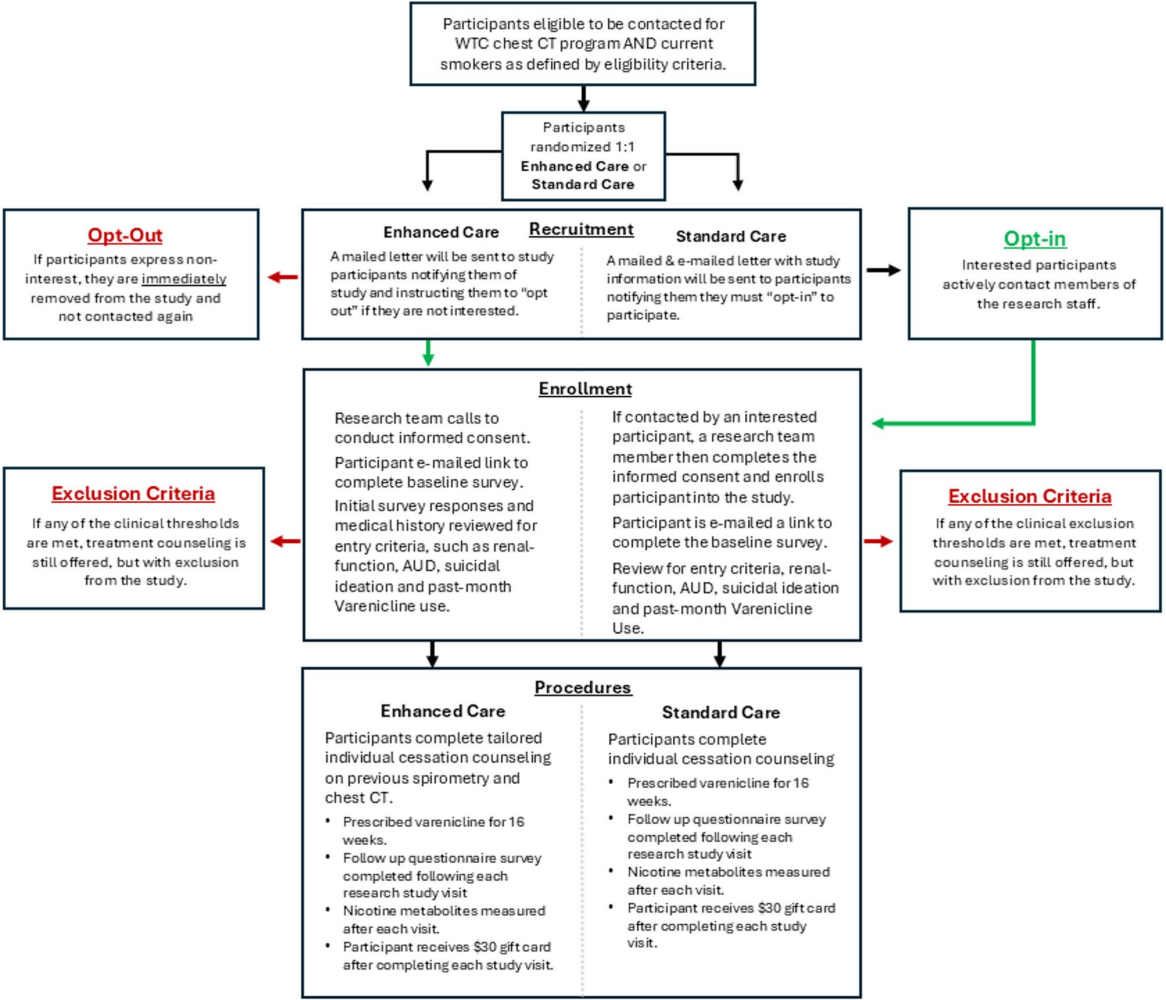
Flow of Participants Through Recruitment, Enrollment, and Study Procedures. **Legend**: This figure illustrates the participant flow for a randomized trial comparing Enhanced Care and Standard Care smoking treatment programs among WTC-exposed responders who smoke. Recruitment strategies differed between groups, with Enhanced Care participants receiving an “opt-out” invitation and Standard Care participants required to “opt-in.” The flowchart outlines the stages of recruitment, enrollment, and study procedures, including eligibility screening, exclusion criteria, counseling sessions, varenicline treatment, and telehealth-based follow-ups. Key study activities, such as research assessments and participant compensation, are also detailed

This trial evaluates the effectiveness of an Enhanced Care intervention (opt-out) versus Standard Care (opt-in) in promoting tobacco cessation among retired FDNY-WTCHP responders who are eligible for LDCT lung cancer screening based on their self-reported smoking history. Using a parallel-arm design, the trial compares these interventions among WTC-exposed responders who currently smoke (Fig. 2). The Enhanced Care intervention includes opt-out enrollment in tobacco treatment, in which participants receive services unless they actively decline, tailored counseling with biofeedback based on LDCT scan and spirometry results, and varenicline with preloading, initiated before the target quit date, along with NRT as needed. In contrast, the Standard Care intervention requires opt-in enrollment and offers standard counseling without personalized biofeedback, and varenicline with preloading, initiated before the target quit date.

All participants follow a standardized treatment protocol that includes varenicline preloading for four weeks prior to the target quit date, followed by 12 weeks of varenicline. Counseling sessions are conducted via telehealth at baseline and weeks 2, 4, 8, 12, and 16, with a final follow-up at week 28. Research assessments are completed online at the same time points: baseline and weeks 2, 4, 8, 12, 16, and 28. Nicotine metabolite urine testing is conducted at Quest Diagnostics labs near participants’ homes. Participants receive a $30 gift card for each research visit they complete, which includes a complete questionnaire, a counselor visit, and a urine test. These measures are designed to enhance participation, adherence, and consistency across both intervention groups, supporting a thorough evaluation of factors influencing cessation outcomes.

The primary objectives of the trial are to compare enrollment rates between the Enhanced Care and Standard Care groups (objective 1), to analyze the impact of sociodemographic and clinical factors on enrollment, and to assess program outcomes: retention, adherence to varenicline and counseling, and tobacco abstinence (objective 2). A summary of study activities is described in Table 2.

**Table 2:** Patient timeline by treatment arm: Enhanced Care and Standard Care

	Enrollment		Baseline (Week 0)		Week 2		Week 4		Week 8		Week 12		Week 16		Week 28	
	S	E	S	E	S	E	S	E	S	E	S	E	S	E	S	E
Enrollment																
**Randomization**: Randomization to treatment group	X	X														
**Opt-out enrollment with counseling**: Participants receive a letter outlining *opt-out* enrollment in the free FDNY-WTCHP Tobacco Cessation Program for CT. Those who do not opt out are proactively contacted by the Research Coordinator to discuss treatment options, explain study procedures, and obtain informed consent. Participants who indicate they do not wish to participate are excluded from the study and are not contacted again, ensuring respect for their preferences.		X														
**Opt-in enrollment with counseling**: Participants are invited to *opt into* the free FDNY-WTCHP Tobacco Cessation Program through a letter describing the program and its benefits, including personalized counseling and support. Enrollment requires the participant to initiate contact, expressing interest in tobacco treatment. The program provides counseling sessions tailored to help participants quit smoking and improve their overall health.	X															
**Treatment visits**																
**Enhanced Counseling**: Telehealth counseling by certified tobacco treatment specialists, focusing on skills per Public Health Service guidelines, personalized biofeedback on LDCT/spirometry results, varenicline support, and lung cancer risk reduction to leverage or enhance motivation.				X		X		X		X		X		X		X
**Standard Counseling**: Certified tobacco treatment specialists provide brief telehealth counseling focused on skill-building in accordance with Public Health Service guidelines, along with varenicline support. Sessions do not include discussions of LDCT/spirometry results.			X		X		X		X		X		X		X	
**Varenicline dosing**: Both groups: 0.5 mg once daily for 3 days, 0.5 mg twice daily for 4 days, then 1 mg twice daily from day 8 onward.			X*	X*	X*	X*	X	X	X	X	X	X	End of treatment			
**Self-administered questionnaires: Both groups -** Patients complete web-based questionnaires			X	X	X	X	X	X	X	X	X	X	X	X	X	X
**Urine collection**																
Both groups - Urine samples will be collected at Quest Diagnostics to measure nicotine metabolite levels			X	X	X	X	X	X	X	X	X	X	X	X	X	X

**E = Enhanced Care S = Standard** Care* Varenicline preloading: Varenicline initiated four weeks prior to target quit date (TQD) followed by 12 weeks of varenicline post-TQD, for a total of 16 weeks of treatment, to reduce pre-treatment smoking enjoyment and increase tobacco abstinence

### Surveys

Baseline and follow-up surveys, conducted within three days of each counseling visit, include comprehensive assessments to monitor smoking behaviors and ensure the continued appropriateness of varenicline use. Baseline surveys cover smoking history, current tobacco use, alternative tobacco use [32, 33], nicotine dependence (via the Fagerstrom Test for Nicotine Dependence) [8], prior quit attempts [34], and cessation motivation [35, 36] and self-efficacy.^36^ Tobacco-related questions include a past-month timeline follow-back (TLFB) calendar [37–40] to capture detailed smoking patterns and daily cigarette consumption. Emotional and psychological domains are assessed using the SF-12 for quality of life [41], the Center for Epidemiologic Studies-Depression (CES-D) scale for depressive symptoms [42, 43], and the Post-traumatic Stress Disorder Checklist (PCL-17) for trauma-related symptoms [44, 45]. Alcohol use is screened using the Alcohol Use Disorders Identification Test (AUDIT) [46], and the Columbia-Suicide Severity Rating Scale (C-SSRS) identifies suicidal ideation or behaviors, triggering immediate intervention when necessary [47]. In cases of a positive C-SSRS screen, the director of mental health services at FDNY is immediately notified via automated text message and email to ensure timely follow-up and support. Follow-up surveys (weeks 2, 4, 8, 12, 16, and 28) focus on tobacco-related questions, including the TLFB calendar and the C-SSRS, to monitor smoking behaviors and ensure participant safety while reducing survey burden. All surveys are completed at home electronically on FDNY-WTCHP’s cloud-based platform. If participants encounter difficulties with technology, surveys will be conducted by a study coordinator by phone to ensure data collection continuity and minimize participant burden.

### Counseling approach

All participants receive seven structured telehealth counseling sessions (via Microsoft Teams) with a certified tobacco treatment specialist. These sessions focus on evidence-based strategies, including problem-solving, skills training, and biofeedback, ensuring fidelity and consistency across study arms while addressing individual needs. Counseling sessions also reinforce adherence to the varenicline regimen (S1).

The counseling approach is informed by a comprehensive checklist adapted from The Manual of Smoking Cessation: A Guide for Counsellors and Practitioners (S2) [48]. Key components include assessing withdrawal symptoms, facilitating action planning, addressing lapse triggers, and reinforcing an identity as someone who has quit smoking. Education on nicotine monitoring and pharmacological aids, combined with biofeedback, ensures participants receive consistent, evidence-based support.

Counseling sessions include building rapport, assessing smoking behaviors and readiness to quit, addressing withdrawal symptoms, and managing barriers to cessation. Participants receive tailored advice on avoiding social smoking cues, restructuring their environment, and developing coping strategies to prevent relapse. Behavioral action plans, goal setting, and progress reviews are integral to each session. Education on the health benefits of quitting, the purpose of nicotine monitoring, and the use of pharmacological aids are also provided. Participants are encouraged to self-record tobacco use, identify reasons for quitting, and engage with social support systems.

Participant’s contact information is verified and updated before each counseling visit. The research team maintains regular communication with participants to optimize retention. These include text messages, e-mail and phone call appointment reminders. Telehealth counseling visits accommodate participant preferences and ensure consistency. Missed visits are rescheduled twice: first on the same day the session was missed and again one week later if the participant has not responded. If the visit is not completed before the next scheduled research visit, it is recorded as missed. Refused sessions are not rescheduled, but counseling is re-offered at the next research visit. Counseling sessions follow the same schedule as research visits, with their timing based on the date of the baseline visit. Missed content may be addressed in a later session or replaced with the scheduled material, as determined by the tobacco treatment specialist.

### Enhanced Care arm

In the Enhanced Care arm, counseling integrates biofeedback from participants’ LDCT and spirometry results to create periods of increased health awareness that may enhance receptivity to behavior change [5, 49]. For individuals with LDCT abnormalities (e.g., nodules, bronchial wall thickening, emphysema, chronic obstructive pulmonary disease (COPD), or fibrosis), counseling highlights the benefits of smoking cessation, including the potential resolution of inflammation in ground-glass nodules and a slower progression of disease. Spirometry results indicating accelerated lung function decline or dysfunction are used to highlight the benefits of quitting in slowing disease progression. Participants with normal findings are counseled on long-term health benefits and preventing future complications. Nicotine metabolite results are incorporated to provide additional biofeedback, reinforcing adherence to the treatment program and titrating nicotine replacement medications when necessary. Nicotine replacement medications are added, if necessary, to attenuate break-through withdrawal symptoms and/or to attain complete abstinence. Enhanced Care sessions are structured to last between 30 and 40 min.

### Standard Care arm

In the Standard Care arm, counseling follows the same structured framework but does not incorporate tailored biofeedback from LDCT or spirometry results. Participants in this arm receive varenicline including preloading. Instead, the sessions focus on general strategies for quitting smoking and maintaining abstinence. Standard care sessions are designed to be brief, typically lasting under 15 min.

### Consistency and fidelity

All counseling sessions are delivered by the same certified tobacco treatment specialist to ensure consistent delivery and minimize differential bias between study arms. To maintain protocol adherence, the tobacco counselor uses a Fidelity Checklist during sessions (S1). This checklist ensures that key components, such as assessing smoking behavior, reviewing medication use, and addressing barriers, are consistently addressed. For Enhanced Care participants, the checklist ensures tailored discussions of LDCT, spirometry, and nicotine metabolite results occur when appropriate. The duration of each session is documented. To maintain impartiality and minimize social desirability bias, the counselor does not perform outcome assessments.

### Pharmacological support

All participants, regardless of study arm, receive a total of 16 weeks of varenicline: preloading for four weeks prior to the participant’s target quit date, followed by 12 weeks of varenicline. Dosing is standard: 0.5 mg daily for three days, followed by 0.5 mg twice daily for days 4 through 7, followed by 1 mg twice daily thereafter. Varenicline preloading is offered to all participants to reduce pre-quit tobacco enjoyment and withdrawal symptoms. Varenicline preloading prior to the target quit date has been shown to reduce the rewarding effects of smoking and increase quit success rates [29]. In both the enhanced and standard care arms, appropriate NRTs are added to varenicline when necessary and substituted in cases of adverse drug events (ADEs) to ensure individual tolerability and safety. Nicotine replacement medications are added, when necessary, to attenuate break-through tobacco withdrawal symptoms and/or to attain complete abstinence.

### Assessment of eligibility and symptom monitoring

Participants are screened at screening and each subsequent research visit for serious or unstable conditions, including psychiatric hospitalization, decompensated cirrhosis, cardiovascular disease, seizure disorder, suicidal ideation or attempts, and adverse reactions to varenicline (S4). Withdrawal symptom severity is assessed using a 0–10 scale for symptoms such as craving, irritability, insomnia, nausea, headaches, and depression. These measures were used to ensure safety and monitor withdrawal effects during the study (S5).

### Measures

For Objective 1, which examines factors related to program enrollment, the primary outcome is FDNY Tobacco Cessation Program enrollment (Yes/No), comparing enrollment rates between Enhanced and Standard Care. Sociodemographic factors (sex, age, race, and occupation (firefighter versus EMS)) and clinical factors (LDCT results, spirometry, PTSD, depression, quality of life, prior quit attempts, and nicotine dependence) are measured as potential influences on program enrollment. Additionally, prior program participation and documented quit attempts in health records are recorded.

For Objective 2, which evaluates tobacco abstinence and participant retention across study arms, the primary outcome is biochemically confirmed 7-day point prevalence abstinence, assessed at both the end of treatment and the conclusion of follow-up. Participants receive requisition forms for urine testing at Quest Diagnostics, to be completed within three days prior to appointments. These tests, pre-paid through the FDNY-WTCHP, provide results to the tobacco counselor in the Enhanced Care arm before sessions. The research team assists participants in locating the nearest Quest Diagnostics facility and scheduling appointments as needed. Biomarkers assessed at baseline and prior to each counseling visit include anabasine, nicotine, and its metabolites (cotinine, 3’-hydroxycotinine, and nornicotine) to monitor smoking status and adherence. Cotinine is used to identify tobacco exposure (cutpoint: 20 ng/mL), while anabasine distinguishes active tobacco use from NRT (cutpoint: 2 ng/mL) [50].

### Analyses

For Objective 1, we will examine factors associated with program enrollment, including sociodemographic and clinical characteristics. Prior program participation and documented quit attempts will be analyzed to assess their influence on cessation program enrollment.

For Objective 2, factors associated with abstinence will be explored, including retention, adherence to counseling sessions, and adherence to varenicline. Pill counts at each visit will assess adherence to prescribed medication (S3). Appointment duration will be recorded as a measure of engagement and intervention adherence. Exploratory analyses will assess the impact of adherence on abstinence rates and examine the influence of Enhanced Care on motivation to quit and abstinence rates.

### Ethics statement

This trial, registered at ClinicalTrials.gov (NCT05997225), was approved by the Montefiore Einstein IRB (#2023–14980). All participants included in the study provide informed consent before participating, and the study follows the Declaration of Helsinki and regulatory guidelines. This manuscript complies with SPIRIT guidelines, which promote transparent reporting of clinical trial protocols, including trial design, enrollment, interventions, and planned outcomes.

## Discussion

This novel study aims to test an Enhanced Care opt-out smoking treatment model tailored to the unique needs of this high-risk cohort, with a focus on overcoming the significant challenges of engaging individuals who smoke in treatment. Addressing these engagement challenges is critical to ensuring the success of cessation efforts, particularly for vulnerable groups like FDNY-WTC responders. Smoking remains the leading preventable cause of death and disease in the U.S., accounting for nearly 500,000 deaths annually, or one in five deaths [51, 52]. Although roughly 55% of people who smoke cigarettes try to quit each year, fewer than 10% succeed [53, 54]. Despite substantial declines in smoking rates among FDNY responders over the past two decades, a subset continues to smoke, facing heightened risks of lung function decline, respiratory disease, and lung cancer.

Among FDNY-WTC responders, the health effects of smoking have been exacerbated by exposure to the WTC disaster. Responders experienced an average 20% loss in lung function following 9/11, with over 20% developing new-onset obstructive airways disease [55]. Studies within the FDNY-WTCHP cohort have demonstrated that lung function decline is most pronounced among individuals who currently smoke, while smoking cessation significantly mitigates this decline [6]. 

The Enhanced Care intervention incorporates several innovative strategies to overcome common barriers to cessation success. The opt-out enrollment model addresses low treatment engagement by enrolling participants unless they actively decline, a tactic proven effective in improving adherence in other chronic disease management contexts, such as colorectal cancer screening [9, 56]. Proactive counseling builds on participants’ LDCT and spirometry findings to deliver personalized, motivational messaging that highlights the immediate and long-term benefits of smoking cessation, leveraging the concept of a “reachable, teachable moment” [5] during periods of heightened health awareness. Such biofeedback interventions have yielded mixed results in motivating behavioral change, with some evidence suggesting potential benefits, particularly when linked to tangible health outcomes [22–27, 57]. No LDCT lung cancer screening program in occupational health has adopted a true opt-out enrollment model, in which services are initiated unless actively declined, despite its alignment with guidelines recognizing high nicotine dependence as a major risk factor. This trial addresses the gap by comparing Standard Care with Enhanced Care to evaluate whether this approach improves enrollment and cessation outcomes in high-risk individuals who smoke.

Additionally, varenicline preloading is offered to all participants to mitigate pre-quit tobacco enjoyment and withdrawal symptoms, supported by evidence from randomized trials showing preloading increases quit rates and reduces cravings [29]. The inclusion of personalized NRT options further supports individualized treatment plans, reflecting best practices recommended by the U.S. Public Health Service [58]. 

The trial faces some potential obstacles. The FDNY-WTCHP cohort is actively followed with numerous opportunities for tobacco cessation treatment and has already achieved substantial success in reducing tobacco use, potentially limiting the magnitude of change detectable with new interventions. The remaining individuals who smoke have been the most challenging to treat. However, the Program’s continued investment in reducing smoking rates to zero underscores the importance of refining and expanding tobacco treatment strategies. Additionally, the design of the Enhanced Care arm may not fully capture its potential impact, as participants must still actively consent to participate despite being proactively contacted by the research team. Nonetheless, the direct comparison of opt-out versus opt-in models is expected to provide valuable insights into strategies for increasing engagement during recruitment that may promote treatment success. The findings may have limited generalizability, as the FDNY-WTCHP population benefits from extensive tobacco cessation resources that are not widely available. Additionally, the enhanced care arm includes additional contact time compared to the standard care arm, which may contribute to differences in outcomes. The bundled intervention, combining opt-out enrollment, biofeedback, and varenicline preloading, presents challenges in isolating the effects of individual components, making it difficult to disentangle the impact of each aspect of the intervention. These factors may limit the feasibility and scalability of the approach in less resource-rich settings. Furthermore, some participants may face challenges with technology, which, along with the virtual nature of the study, could pose additional barriers to engagement and adherence.

This trial has the potential to provide several important benefits, including mitigating the burden of smoking-related diseases such as COPD, cancer, and cardiovascular disease. Additionally, the Enhanced Care model may contribute to improved quality of life and reduced healthcare costs among responders [59]. Findings could inform occupational and LDCT lung cancer screening programs by integrating tobacco treatment initiatives, which can expand access to treatment and counter the misconception that screening eliminates the need to quit smoking [60]. This integration is especially relevant given evidence suggesting that tobacco treatment interventions significantly enhance the cost-effectiveness of LDCT lung cancer screening by reducing smoking-related disease incidence [61, 62].

This study seeks to redefine tobacco treatment by engaging high-risk individuals who smoke and have struggled with traditional methods. By combining opt-out enrollment, biofeedback from LDCT and spirometry, and varenicline preloading, the intervention aligns with CMS recommendations for integrating tobacco counseling into LDCT lung cancer screening [43] and is novel in its application to an occupational cohort. It addresses a critical research gap, targeting a challenging-to-treat population with significant tobacco exposure and health risks. If successful, the Enhanced Care model could reduce smoking-related health burdens and offer scalable strategies to improve treatment outcomes in both occupational and broader populations.

## Supplementary Information

Below is the link to the electronic supplementary material.

Supplementary Material 1

## Data Availability

Data sharing is not applicable to this article as no datasets were generated or analyzed during the current study.

## Abbreviations

9/11: September 11 2001
ADE: Adverse Drug Events
AUDIT: Alcohol Use Disorders Identification Test
CESD: Center for Epidemiologic Studies-Depression
CMS: Centers for Medicare and Medicaid Services
COPD: Chronic Obstructive Pulmonary Disease
CSSRS: Columbia-Suicide Severity Rating Scale
EMS: Emergency Medical Services
eGFR: Estimated Glomerular Filtration Rate
FDNY: Fire Department of the City of New York
FDNY-WTCHP: Fire Department of the City of New York World Trade Center Health Program
IRB: Institutional Review Board
LDCT: Low-Dose Computed Tomography
NCCN: National Comprehensive Cancer Network
NIOSH: National Institute for Occupational Safety and Health
NRT: Nicotine Replacement Therapy
OAD: Obstructive Airways Disease
PCL-17: Post-traumatic Stress Disorder Checklist
RCT: Randomized Controlled Trial
SF-12: Short Form-12 Health Survey
TLFB: Timeline Follow-Back
WTC: World Trade Center
