# Supplementary material for: Design of a randomized tobacco cessation trial among FDNY World Trade Center responders in a lung cancer screening program
Source: Addict Sci Clin Pract. 2025 Aug 5;20:62. doi: 10.1186/s13722-025-00598-3 (PMC12323262; doi:10.1186/s13722-025-00598-3)
Supplement: Supplementary file 1 — Supplementary Material 1 [file 13722_2025_598_MOESM1_ESM.docx]

**Supplemental information**

**Figure S1:** Fidelity checklist

**Treatment Fidelity Checklist**

Participant ID#:

Treatment Arm:

Today’s date:

Target quit date:

| **Delivery of Intervention Components (protocol requirements)** | | | | |
| --- | --- | --- | --- | --- |
| **In this session did you:** | | | | |
| 1. Assess for clinical instability? | YES | NO |  |  |
| 1. Assess level of smoking & progress | YES | NO | Partially | NA |
| 1. Review medication use (if appropriate) | YES | NO | Partially | NA |
| 1. Conducted pill count/took screen shot of pack. ***Follow-up sessions, only (visits 2-7)*** | YES | NO | Partially | NA |
| 1. Bridge from previous sessions (counselor summaries and follow up on goals etc.) ***Follow-up sessions, only (visits 2-7)*** | YES | NO | Partially | NA |
| 1. Delivered treatment component according to readiness to quit | YES | NO | Partially | NA |
| 1. Document quit date ***Baseline only*** | YES | NO | Partially | NA |
| 1. Discuss behavioral action plan | YES | NO | Partially | NA |
| 1. Assess and record adverse events | YES | NO | Partially | NA |
| 1. Start time (Counseling should begin after pill count and introductions) | XX:XX AM/PM | | | |

**Supplemental Figures**

**Figure S2: Enhanced Care Visit checklist**

| **Item** | **Completed** |
| --- | --- |
| Strengthen ex-smoker identity |  |
| Elicit patient views |  |
| Measure Nicotine Metabolites |  |
| Give options for additional and later support |  |
| Provide rewards contingent on successfully stopping smoking |  |
| Advice on changing routine |  |
| Facilitate relapse prevention and coping |  |
| Ask about previous experiences with stop smoking medications |  |
| Advice on stop smoking medication(s) |  |
| Advise on/facilitate use of social support |  |
| Summarize information/confirm patient decisions |  |
| Provide reassurance |  |
| Boost motivation and self-efficacy |  |
| Provide information on withdrawal symptoms |  |
| Explain the purpose of CO/ Nicotine monitoring |  |
| Use reflective listening |  |
| Help patients to obtain medication |  |
| Advice on avoidance of social cues for smoking |  |
| Advice on environmental restructuring |  |
| Assess current and past smoking behavior |  |
| Assess current readiness and ability to quit |  |
| Assess history of quit attempts |  |
| Assess withdrawal symptoms |  |
| Build general rapport |  |
| Elicit and answer questions |  |
| Emphasize choice |  |
| Explain expectations regarding treatment program |  |
| Explain the importance of abrupt cessation |  |
| Facilitate action planning/help identify lapse triggers |  |
| Facilitate barrier identification and problem solving |  |
| Facilitate goal setting |  |
| Identify reasons for wanting and not wanting to stop smoking |  |
| Offer/direct toward appropriate written materials |  |
| Prompt commitment from the patient |  |
| Prompt review of goals |  |
| Prompt self-recording of tobacco use |  |
| Provide feedback on current behaviors |  |
| Provide information on consequences of tobacco and tobacco abstinence |  |
| Provide normative information about others’ behavior and experiences |  |
| Rewards contingent on effort or progress |  |
| Set tasks / homework |  |
| Tailor interaction appropriately |  |

**Figure S3: Pill Counting checklist**

FDNY PILL COUNT SHEET

*Ask the following questions and make necessary changes in tables below:*

1) Are you still taking this medication? Yes No

2) Since your last research visit, did you lose any pills, give any pills away, or take pills out of any of these containers that you did not take?

Yes No If yes, how many?

Date medication initiated: Click or tap to enter a date.

Date pack #1 started: Date pack #2 started:

Date pack #3 started: Date pack #4 started:

| ***Visit**** | ***Visit Date*** | ***Pack/bottle #*** | ***Total quantity*** | ***Pills Taken*** | ***# of Pills Remaining*** | ***Other notes*** |
| --- | --- | --- | --- | --- | --- | --- |
| *2* |  |  |  |  |  |  |
|  |  |  |  |  |  |  |
| *3* |  |  |  |  |  |  |
|  |  |  |  |  |  |  |
| *4* |  |  |  |  |  |  |
|  |  |  |  |  |  |  |
| *5* |  |  |  |  |  |  |
|  |  |  |  |  |  |  |
| *6* |  |  |  |  |  |  |
|  |  |  |  |  |  |  |
| *7* |  |  |  |  |  |  |
|  |  |  |  |  |  |  |

Note: The certified tobacco treatment specialist electronically maintains the pill count log.

**Figure S4: Assessing serious or unstable disease**

| **Has the individual had:** | YES | NO |
| --- | --- | --- |
| Psychiatric hospitalization in the past year | ​​☐​ | ​​☐​ |
| Decompensated cirrhosis | ​​☐​ | ​​☐​ |
| Serious cardiovascular disease | ​​☐​ | ​​☐​ |
| History of seizure disorder | ​​☐​ | ​​☐​ |
| Current suicidal ideation | ​​☐​ | ​​☐​ |
| History of suicide attempt in the past year | ​​☐​ | ​​☐​ |
| Previously had side effects/ adverse events associated with varenicline | ​​☐​ | ​​☐​ |

***Figure S5:*** *Symptom checklist*

*Study ID:*  *Date:*

| Symptom | 0 (absent) | 1 | 2 | 3 | 4 | 5 | 6 | 7 | 8 | 9 | 10 (Severe) |
| --- | --- | --- | --- | --- | --- | --- | --- | --- | --- | --- | --- |
| Cigarette craving | ​​☐​ | ​​☐​ | ​​☐​ | ​​☐​ | ​​☐​ | ​​☐​ | ​​☐​ | ​​☐​ | ​​☐​ | ​​☐​ | ​​☐​ |
| Irritability/impatience | ​​☐​ | ​​☐​ | ​​☐​ | ​​☐​ | ​​☐​ | ​​☐​ | ​​☐​ | ​​☐​ | ​​☐​ | ​​☐​ | ​​☐​ |
| Insomnia/sleeplessness | ​​☐​ | ​​☐​ | ​​☐​ | ​​☐​ | ​​☐​ | ​​☐​ | ​​☐​ | ​​☐​ | ​​☐​ | ​​☐​ | ​​☐​ |
| Shakiness/tremors | ​​☐​ | ​​☐​ | ​​☐​ | ​​☐​ | ​​☐​ | ​​☐​ | ​​☐​ | ​​☐​ | ​​☐​ | ​​☐​ | ​​☐​ |
| Increased hunger | ​​☐​ | ​​☐​ | ​​☐​ | ​​☐​ | ​​☐​ | ​​☐​ | ​​☐​ | ​​☐​ | ​​☐​ | ​​☐​ | ​​☐​ |
| Over-eating | ​​☐​ | ​​☐​ | ​​☐​ | ​​☐​ | ​​☐​ | ​​☐​ | ​​☐​ | ​​☐​ | ​​☐​ | ​​☐​ | ​​☐​ |
| Headaches | ​​☐​ | ​​☐​ | ​​☐​ | ​​☐​ | ​​☐​ | ​​☐​ | ​​☐​ | ​​☐​ | ​​☐​ | ​​☐​ | ​​☐​ |
| Nausea/abdominal pain | ​​☐​ | ​​☐​ | ​​☐​ | ​​☐​ | ​​☐​ | ​​☐​ | ​​☐​ | ​​☐​ | ​​☐​ | ​​☐​ | ​​☐​ |
| Mouth sores/bleeding gums | ​​☐​ | ​​☐​ | ​​☐​ | ​​☐​ | ​​☐​ | ​​☐​ | ​​☐​ | ​​☐​ | ​​☐​ | ​​☐​ | ​​☐​ |
| Constipation | ​​☐​ | ​​☐​ | ​​☐​ | ​​☐​ | ​​☐​ | ​​☐​ | ​​☐​ | ​​☐​ | ​​☐​ | ​​☐​ | ​​☐​ |
| Difficulty concentrating | ​​☐​ | ​​☐​ | ​​☐​ | ​​☐​ | ​​☐​ | ​​☐​ | ​​☐​ | ​​☐​ | ​​☐​ | ​​☐​ | ​​☐​ |
| Chest pain/tightness | ​​☐​ | ​​☐​ | ​​☐​ | ​​☐​ | ​​☐​ | ​​☐​ | ​​☐​ | ​​☐​ | ​​☐​ | ​​☐​ | ​​☐​ |
| Drowsiness | ​​☐​ | ​​☐​ | ​​☐​ | ​​☐​ | ​​☐​ | ​​☐​ | ​​☐​ | ​​☐​ | ​​☐​ | ​​☐​ | ​​☐​ |
| Sweating | ​​☐​ | ​​☐​ | ​​☐​ | ​​☐​ | ​​☐​ | ​​☐​ | ​​☐​ | ​​☐​ | ​​☐​ | ​​☐​ | ​​☐​ |
| Heart palpitations | ​​☐​ | ​​☐​ | ​​☐​ | ​​☐​ | ​​☐​ | ​​☐​ | ​​☐​ | ​​☐​ | ​​☐​ | ​​☐​ | ​​☐​ |
| Abnormal dreams | ​​☐​ | ​​☐​ | ​​☐​ | ​​☐​ | ​​☐​ | ​​☐​ | ​​☐​ | ​​☐​ | ​​☐​ | ​​☐​ | ​​☐​ |
| Dizziness/lightheadedness | ​​☐​ | ​​☐​ | ​​☐​ | ​​☐​ | ​​☐​ | ​​☐​ | ​​☐​ | ​​☐​ | ​​☐​ | ​​☐​ | ​​☐​ |
| Depression/sadness | ​​☐​ | ​​☐​ | ​​☐​ | ​​☐​ | ​​☐​ | ​​☐​ | ​​☐​ | ​​☐​ | ​​☐​ | ​​☐​ | ​​☐​ |
| Rash | ​​☐​ | ​​☐​ | ​​☐​ | ​​☐​ | ​​☐​ | ​​☐​ | ​​☐​ | ​​☐​ | ​​☐​ | ​​☐​ | ​​☐​ |
